# Discovery of Novel and Highly Potent Inhibitors of SARS CoV-2 Papain-Like Protease Through Structure-Based Pharmacophore Modeling, Virtual Screening, Molecular Docking, Molecular Dynamics Simulations, and Biological Evaluation

**DOI:** 10.3389/fphar.2022.817715

**Published:** 2022-02-21

**Authors:** Xiaoyan Tian, Quanfeng Zhao, Xiaohong Chen, Zhe Peng, Xiaodan Tan, Qin Wang, Lin Chen, Yang Yang

**Affiliations:** ^1^ Department of Pharmacology, Chongqing Health Center for Women and Children, Chongqing, China; ^2^ Department of Pharmacy, Southwest Hospital, First Affiliated Hospital to TMMU, Third Military Medical University (Army Medical University), Chongqing, China; ^3^ Department of Pharmacology, Chongqing Hospital of Traditional Chinese Medicine, Chongqing, China

**Keywords:** SARS CoV-2, papain-like protease, pharmacophore modeling, molecular docking, inhibitor

## Abstract

**Background and Objective:** COVID-19 has struck our society as a great calamity, and the need for effective anti-viral drugs is more urgent than ever. Papain-like protease (PLpro) of SARS CoV-2 plays important roles in virus maturation, dysregulation of host inflammation, and antiviral immune responses, which is being regarded as a promising druggable target for the treatment of COVID-19. Here, we carried out a combined screening approach to identify novel and highly potent PLpro inhibitors for the treatment of COVID-19.

**Methods:** We used a combined screening approach of structure-based pharmacophore modeling and molecular docking to screen an in-house database containing 35,000 compounds. SARS CoV-2 PLpro inhibition assay was used to carry out the biological evaluation of hit compounds. Molecular dynamics (MD) simulations were conducted to check the stability of the PLpro-hit complexes predicted by molecular docking.

**Results:** We found that four hit compounds showed excellent inhibitory activities against PLpro with IC_50_ values ranging from 0.6 to 2.4 μM. Among them, the most promising compound, hit 2 is the best PLpro inhibitor and its inhibitory activity was about 4 times higher than that of the positive control (GRL0617). The study of MD simulations indicated that four hits could bind stably to the active site of PLpro. Further study of interaction analysis indicated that hit 2 could form hydrogen-bond interactions with the key amino acids such as Gln269 and Asp164 in the PLpro-active site.

**Conclusion:** Hit 2 is a novel and highly potent PLpro inhibitor, which will open the way for the development of clinical PLpro inhibitors for the treatment of COVID-19.

## Introduction

The pandemic of coronavirus disease 2019 (COVID-19) is causing millions of deaths and immeasurable damage to the global economy (Morens and Fauci, 2020). As of March 15, 2021, the COVID-19 pandemic has resulted in 120,105,958 confirmed cases and 2,657,629 deaths, making it the most serious pandemic since the 1918 Spanish flu (Ma et al., 2021). The novel severe acute respiratory syndrome coronavirus 2 (SARS-CoV-2) is the etiological agent responsible for the pandemic (Wu et al., 2020; Zhou et al., 2020). More urgently, SARS-CoV-2 is highly infectious and is still spreading rapidly across the world. Although some existing anti-viral drugs such as hydroxychloroquine, lopinavir, and chloroquine are recommended to treat seriously ill older patients, it is hard to verify the safety profile, effective role, and adverse effects of these drugs due to the lack of complete data from large randomized clinical trials (RCTs) (Duan et al., 2020; Jafari et al., 2020). Therefore, anti-SARS-CoV-2 drugs are urgently needed.

To date, there have been the major worldwide outbreaks of coronaviruses in the 21st century: MERS-CoV, SARS-CoV, and SARS-CoV-2 (Lu et al., 2020). With respect to novelty, SARS-CoV-2 is different from the two other coronaviruses, MERS-CoV and SARS-CoV. The SARS-CoV-2 is more distant from the coronavirus caused by MERS-CoV, and it is similar to SARS-CoV (Báez-Santos et al., 2015; Lu et al., 2020). In previous works, the coronaviral proteases of SARS-CoV and MERS-CoV, including papain-like protease (PLpro) and 3-chymotrypsin-like protease (3CLpro), were regarded as essential proteolytic enzymes involved in the coronaviral replication process (Clemente et al., 2020). The multiple cellular functions of PLpro are of great significance. First, as cysteine proteases, PLpro is responsible for cleaving polyprotein to generate 16 non-structural proteins (NSPs) that assemble to form the replicase complex to direct the replication and transcription process of the viral genome (Li and Lin, 2013; Mielech, Kilianski et al., 2014; Clemente et al., 2020). Besides, PLpro also has deubiquitinating (DUB) and deISGylating activities that involve in stripping the ubiquitin and IFN-stimulated genes 15 (ISG15) from host proteins to regulate innate immune responses (Ratia et al., 2008; Harty et al., 2009; Mielech, Chen et al., 2014; Zhou et al., 2017; Clasman et al., 2020). In view of the critical role of PLpro in virus invasion, it has been considered as an important target for the treatment in various coronaviruses. In previous studies, Ratia et al. have discovered a noncovalent lead inhibitor GRL0617 through high-throughput screening (HTS) that could inhibit the replication process of SARS-CoV in Vero E6 cells with no cytotoxicity (Lin et al., 2018). Moreover, Lin et al. also verified that the drug Disulfiram used for alcohol aversion therapy had a competitive inhibitory effect on PLpro of MERS-CoV and SARS-CoV, and effectively inhibit their proliferation (Cho et al., 2013). Similarly, Cho et al. extracted and isolated 12 types of flavonoids from a plant called Paulownia tomentosa, which showed potent inhibitory effects on PLpro of SARS-CoV (Bhattacharyya et al., 2017). Although several PLpro inhibitors of SARS-CoV were reported, there is yet little effective drug inhibition of SARS CoV-2. Therefore, the requirement of rapidly screening novel and high-affinity inhibitors specifically targeting PLpro of SARA-CoV-2 is becoming more and more urgent.

Compared with the traditional drug discovery strategies, computer-aided drug design (CADD) not only saves time but also helps to cut costs of designing drugs (McInnes, 2007; Murgueitio et al., 2012), which is regarded as a fast and reliable tool in the pharmaceutical industry. Structure-based virtual screening that combines structure-based pharmacophore modeling and molecular docking is one of the methods used in CADD and it enables screening of many compounds in a relatively short time compared to the high throughput screening via laboratory experiments (McInnes, 2007; Yang et al., 2020). The previous studies have successfully identified kinds of novel and effective inhibitors by using such a virtual screening strategy (Yang et al., 2020; Zhou et al., 2021).

In this work, a structure-based pharmacophore model was constructed based on the resolved crystal structure of PLpro. The model was used as a 3D query to retrieve potential PLpro hits from an in-house database by a root-mean-square distance (RMSD) value between the query features and their matching ligand annotation points, which is the degree of consistency with the pharmacophore model. Subsequently, the retrieved hits were docked into PLpro-binding site. Finally, four novel hits with lower RMSD values and better docking scores were identified and were further tested in the enzyme inhibition assay. These compounds showed significant inhibitory activity toward SARS CoV-2 PLpro. Among them, hit 2 and hit 4 inhibited PLpro with IC_50_ values in the low micromolar range. Biological experiments confirmed that hit 2 and 4 were identified as the novel and potent SARS CoV-2 PLpro inhibitors with potential for the treatment of COVID-19.

## Methods

### Pharmacophore Model Generation and Validation

A high-resolution complex structure of SARS CoV-2 PLpro in complex with a highly effective SARS-CoV PLpro inhibitor GRL0617 (PDB code: 7CMD) obtained from the Protein Data Bank (PDB) was selected to determine the pharmacophore features of the active site according to the following criteria: 1) The organism of selected protein crystal structure should be SARS CoV-2 rather than other species. The organism of the PLpro structure is SARS CoV-2. 2) One of the quality indexes of protein crystal structure is resolution, which represents the uncertainty of atomic position in crystal structure model. When there are many crystal structures available, we choose the structure with high resolution (that is, the crystal structure with small resolution value). In general, a resolution of less than 3 Å is good enough for pharmacophore modeling. To establish an excellent pharmacophore model, the PLpro crystal structure has a high-resolution of 2.59 Å. 3) The selected crystal structure of protein should contain the active pocket; the crystal structure of PLpro protein contains the active binding pocket. 4) The key amino acids in the active pocket of protein should not be missing; the key amino acids such as Gln269, Asp164, Tyr264, Leu162, Pro248, and Pro247 that are necessary for the inhibitor binding exist in the active pocket of the PLpro crystal structure. The Prepare Protein tool of MOE software (Chemical Computing Group Inc., Montreal, Quebec, Canada) was used to prepare the complex structure of SARS-CoV PLpro including hydrogenation, deletion of water molecules, and energy optimization. Then, Ligand Interactions tool of the Molecular Operating Environment (MOE, Chemical Computing Group Inc., Montreal, Quebec, Canada) was used to analyze the interactions between GRL0617 and key amino acids of the PLpro active pocket. According to the protein-ligand interactions, the Pharmacophore Query editor of MOE was further applied to generate the pharmacophore features of PLpro. According to the chemical properties of the PLpro active site, hydrogen-bond acceptor (Acc), hydrogen-bond donor (Don), and aromatic center (Aro) features were selected in the pharmacophore scheme. The resulting pharmacophore model includes the crucial pharmacophore characteristics, which represent the key interaction points with the important residues in the PLpro active site.

The Güner–Henry (GH) test score is used to quantify the model selectivity. The virtual screening of a testing set database was carried out using the pharmacophore search protocol of MOE. The hit lists were analyzed based on the following formula:

$$GH=\left(\frac{Ha\left(3A+Ht\right)}{4HtA}\right)\left(1-\frac{\left(Ht-Ha\right)}{\left(D-A\right)}\right)$$

Statistical parameters including total molecules in database (D), total hits (Ht), active hits (Ha), and goodness of hit score (GH) were calculated. The GH score ranges from 0 to 1, which indicates a null model and an ideal model.

### Virtual Screening

An in-house database contains 35,000 compounds (Niu et al., 2015; Zhou, Tang et al., 2019). First, the conformation import protocol of MOE is used to convert the 2D structure of each molecule to a 3D structure using the MMFF94× force field. In addition, hydrogen atoms are added, partial charges are computed, and multiple conformations per molecule were generated. Then, the pharmacophore search protocol of MOE was used to perform virtual screening based on the generated pharmacophore model. Hit molecules (hit list) can be sorted according to the root of the mean square distance (RMSD) values between the query features of the model and their matching ligand annotation points.

### In Silico ADME Studies

ADMETlab web server (https://admetmesh.scbdd.com/) was used to predict the ADME properties of selected hits (Debnath et al., 2020). The molecular weight (mol_MW), number of hydrogen bond acceptors (nHA), number of hydrogen bond donors (nHD), the logarithm of the n-octanol/water distribution coefficient (logP), apparent permeability coefficient (Madin-Darby Canine Kidney cells, MDCK), human intestinal absorption (HIA), volume distribution (VD), CYP2D6, CYP2C9, and half-life (T_1/2_) were evaluated. Their numerical values were interpreted by the qualitative units based on the ADMETlab server explanation.

### Molecular Docking Experiments

The crystal structure of SARS CoV-2 papain-like protease (PDB ID: 7CMD) was obtained from the PDB database (Osipiuk et al., 2021). Energy minimization was performed using the OPLS-AA force field and hydrogen atoms were added to the protein. The triangle matcher docking protocol of MOE was used to carry out docking of the ligands. The resulting poses are then scored using the docking scoring function.

### Molecular Dynamics Simulations

Based on the docking results, protein-hit complexes were subjected to MD simulation studies using the Groningen Machine for Chemicals Simulations (GROMACS) package under periodic boundary conditions for molecules in order to understand the protein ligand interaction evolved with time inside the active pocket (Dehury, Somavarapu et al., 2020; Dehury, Raina et al., 2020). ACPYPE server was used for the generation of topologies-coordinate files of the selected hits. The native protein topology parameter files were created using Amber99sb-ildn force field. The simple point charge (SPC) water molecules within a cubic period box of 1.0 nm distance were used to solvate the protein-hit complex system and the free protein. The counter ions were added to the system to keep it neutral followed by energy minimization of a complex system using the steepest descent method with a tolerance of 10 kJ/mol/nm. NVT ensemble via the Nose-Hoover method at 310 K was employed to maintain the temperature of each system, while the pressure was equilibrated using an NPT ensemble at 1 bar with the Parinello–Rahman algorithm Finally, the total production run for MD simulations was carried out for 50 ns. Periodic boundary conditions were applied to all simulations, and bonds involving hydrogen atoms were constrained using the linear-constraint-solving (LINCS) algorithm. Trajectory data were saved at time intervals of 100 ps. The molecular dynamics stability parameters were analyzed using toolkits of GROMACS and VMD version1.9.3. The 2D plots were generated using Graphpad Prism 7. MOE was used for protein-hit complexes interaction analysis and image rendering. To explore the conformational heterogeneity in the ensemble of protein-hit complexes structures that visited most frequently along the trajectory, we employed the clustering approach employed in the GROMOS clustering algorithm with a cutoff of 0.18 nm to extract a representative structure. To determine the evolution of the secondary structural elements in the complex during MD simulations, the gmx do_dssp program was used. Hydrogen bonds formed between specific amino acid residues and the hit was analyzed utilizing the gmx hbond. The distance between the key interacting pairs was computed using the gmx distance.

### SARS CoV-2 PLpro Inhibition Assay

Inhibition assays were carried out in a 96-well plate format in triplicate at room temperature. Reactions containing various concentrations of inhibitor (0–500 µM) and PLpro enzyme (0.3 µM) in Tris-HCl pH 7.3, 1 mM EDTA was incubated for approximately 5 min. Then, reactions were initiated with LKGGAMC probe substrate (40 µM), shaken linearly for 5 s, and then measured continuously for fluorescence emission intensity (*λ*_excitation_ = 364 nm; *λ*_emission_ = 440 nm) on a Synergy Neo2 Hybrid. IC_50_ values were calculated using nonlinear regression analysis in the GraphPad Prism software (GraphPad, San Diego, CA).

## Results and Discussion

### Pharmacophore Model Generation and Validation

**FIGURE 1:**
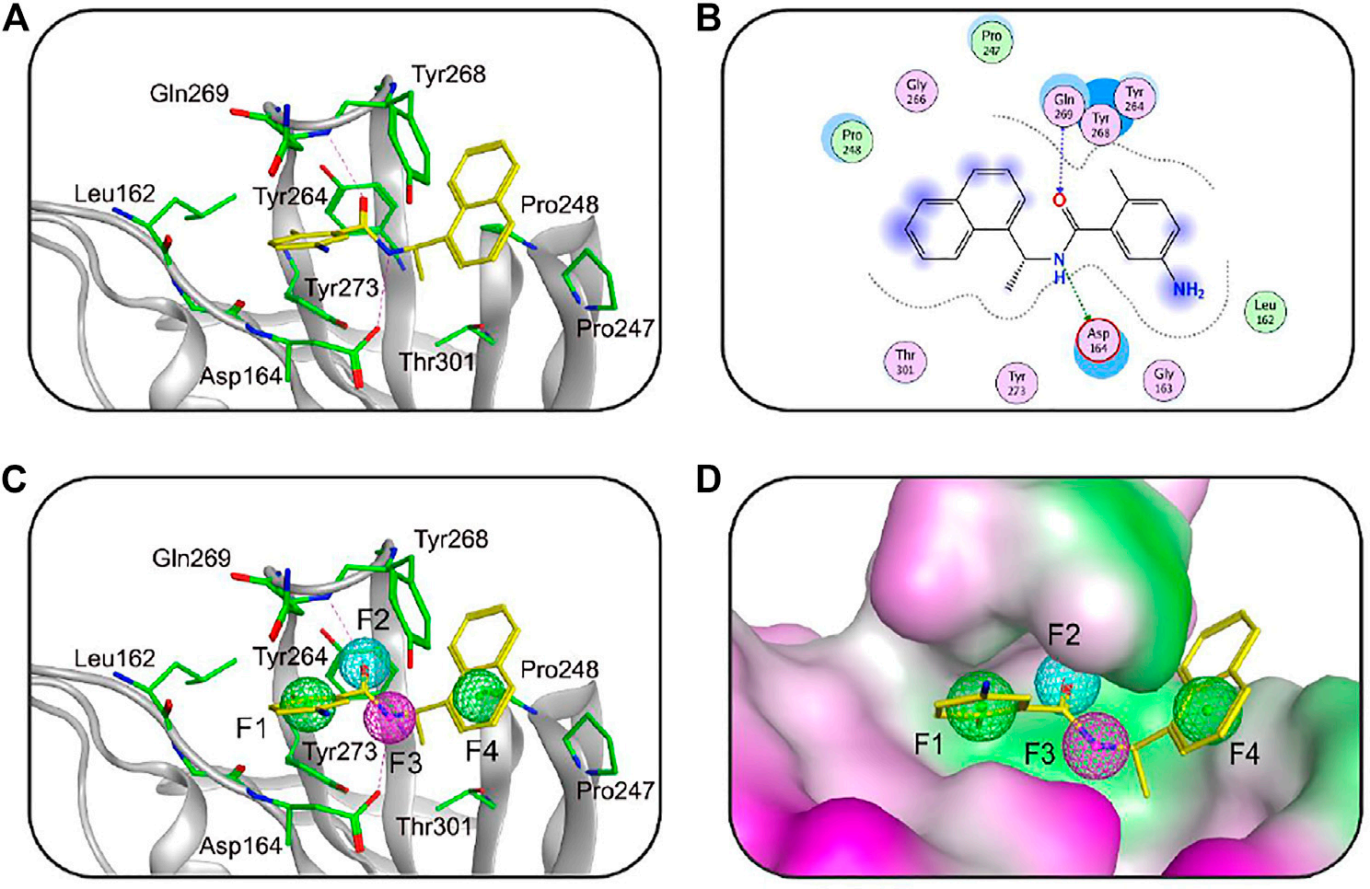
**(A)** The three-dimensional (3D) interaction diagram of the binding site of SARS-CoV-2 PLpro (PDB ID: 7CMD) with GRL0617. The active-site residues and GRL0617 are shown in stick form. The hydrogen-bond network with residues is indicated by red dotted lines. **(B)** 2D interaction diagram of the binding site of SARS-CoV-2 PLpro with GRL0617. **(C)** The generated 3D pharmacophore model at the active site of PLpro. **(D)** Closeup stereo view of the pharmacophore model. Pharmacophore features are color-coded: green, two aromatic features (F1 and F4); cyan, one hydrogen-bond acceptor feature (F2); purple, one hydrogen-bond donor feature (F3).

The structure-based pharmacophore model has become an important tool in drug discovery (Yang et al., 2020; Zhou et al., 2021). Previous studies have shown that MOE can be applied to obtain the pharmacophore features on the ligand binding to the target protein by analyzing the protein-ligand interaction of its crystal structure (Zhou et al., 2021). Recently, Finnin et al. have described the X-ray crystal structure of the SARS-CoV-2 PLpro in complex with a highly effective PLpro inhibitor GRL0617 (PDB code: 7CMD), providing important information of binding requirements for this class of PLpro (Osipiuk et al., 2021). The aim of this study is to use this crystal structure to construct a 3D pharmacophore model of the active site of SARS-CoV-2 PLpro that could enhance the identification process of new chemical scaffolds as potential PLpro inhibitors. The Ligand Interactions tool of MOE was used to analyze the interaction of GRL0617 with key amino acids of the PLpro active site. Based on the analysis of the protein-ligand interaction (Figures 1A,B), the Pharmacophore Query editor of MOE was further applied to generate the pharmacophore model of PLpro (namely, PLpro2-model) containing two aromatic features (F1 and F4), one hydrogen-bond acceptor feature (F2), and one hydrogen-bond donor feature (F3). As shown in Figures 1C,D, these pharmacophore features occupied the PLpro-active site surrounded by the amino acids including Gln269, Asp164, Tyr264, Leu162, Pro248, and Pro247. The features of F2 and F3 matching with oxygen and nitrogen atoms of GRL0617, respectively, exhibited hydrogen-bond interactions with Gln269 and Asp164 in the active site of PLpro, while the aromatic features of F1 and F4 matching with its aromatic rings formed important hydrophobic interactions with hydrophobic residues such as Tyr264, Leu162, Pro248, and Pro247. These results show that the pharmacophore features of the PLpro-model can correctly reflect the interactions with key residues of the PLpro active pocket, further indicating the validity of the PLpro-model.

The GH score method was applied to evaluate the ability of our pharmacophore model to discriminate known active inhibitors against a great number of decoy compounds in the database (Zhou, Di et al., 2019). The decoy set contains a total of 1320 molecules, including 7 active inhibitors (collected from the reported literatures (Osipiuk et al., 2021)). The PLpro-model was applied to screen the decoy set by using the Pharmacophore Search tool. As shown in Table 1, the pharmacophore-based screening results were analyzed using all the calculated statistical parameters, such as the total hits (*Ht*), active hits (*Ha*), enrichment factor (*E*), false negatives, false positives, and goodness of hit score (*GH*). According to the validated results, 9 molecules were retrieved, and the hit rate was 78%. In addition, the SARS CoV-2 PLpro-model showed the enrichment factor of 147 and the GH score of 0.83, indicating the good quality of our model. The GH score method validated that PLpro-model can retrieve potent PLpro inhibitors from the database.

**TABLE 1:** Pharmacophore model validation using GH score method.

Serial no.	Parameter	Pharmacophore model
1	Total molecules in database (*D*)	1320
2	Total number of actives in database (*A*)	7
3	Total hits (*Ht*)	9
4	Active hits (*Ha*)	7
5	% Yield of actives[(*Ha*/*Ht*) × 100]	78%
6	% Ratio of actives [(*Ha*/*A*) × 100]	100%
7	Enrichment factor (*E*) [(*Ha* × *D*)/(*Ht* × *A*)]	147
8	False negatives [*A*- *Ha*]	0
9	False positives [*Ht* - *Ha*]	2
10	Goodness of hit score (*GH*)	0.83

### Database Screening

**FIGURE 2:**
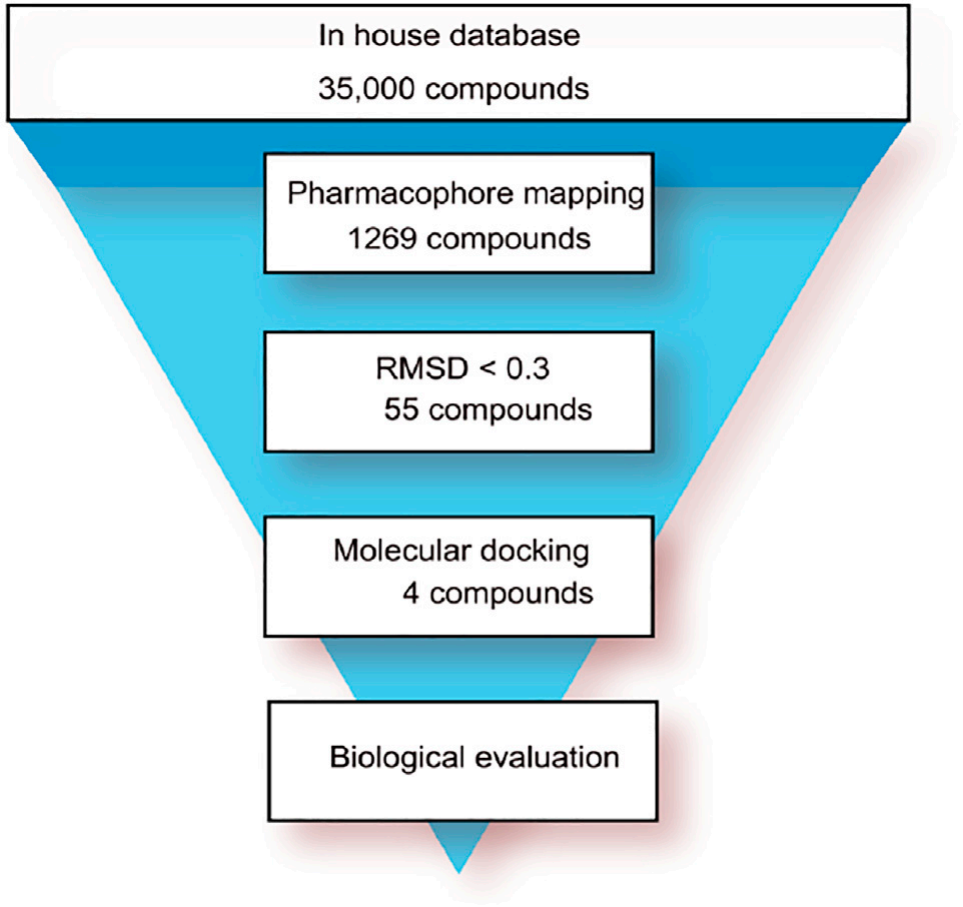
A workflow overview of pharmacophore modeling and selection of hit compounds.

**FIGURE 3:**
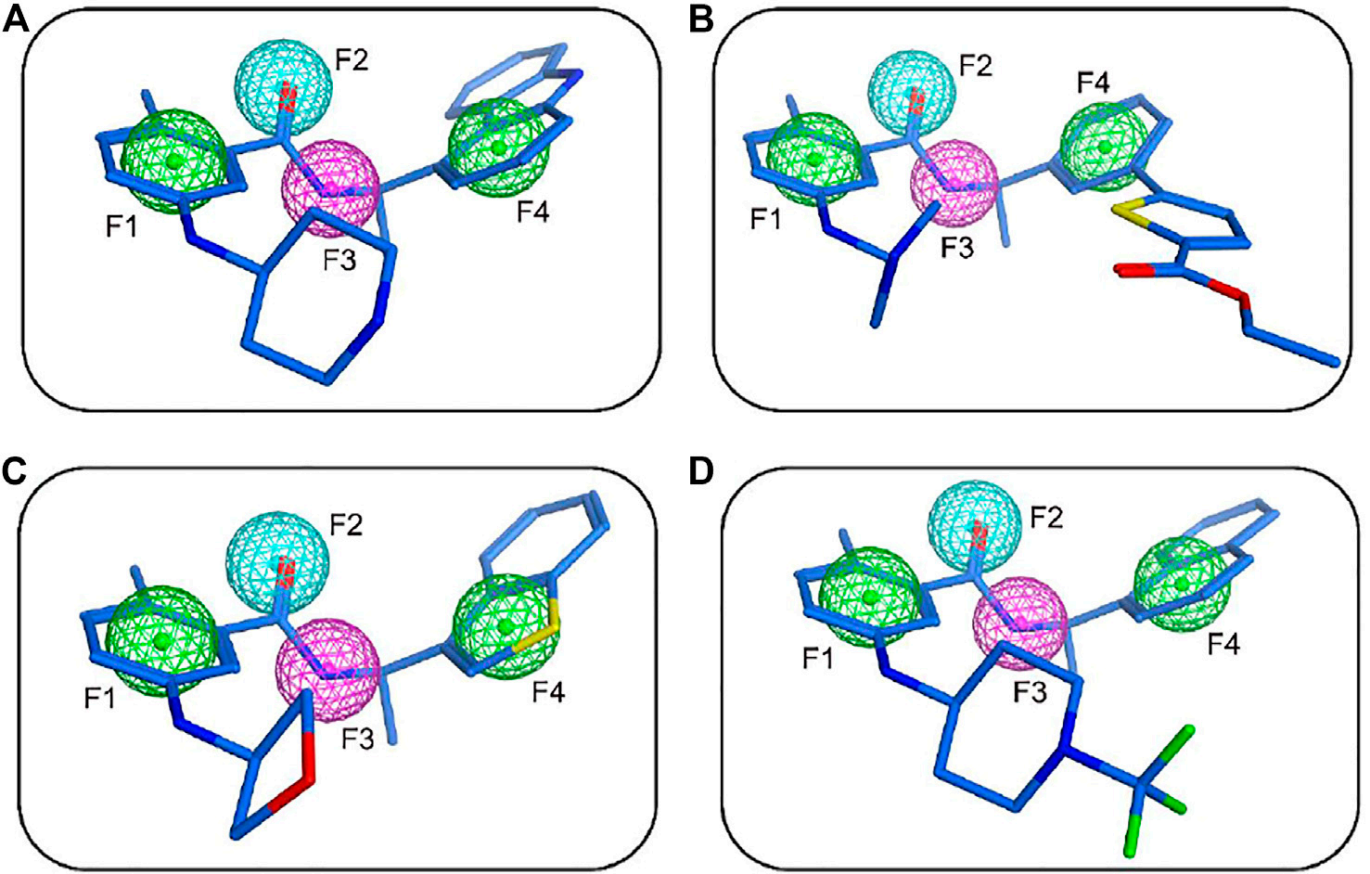
The mapping of the PLpro-model with hit 1 **(A)**, hit 2 **(B)**, hit 3 **(C)**, and hit 4 **(D)**. Hits are depicted as blue stick form. Pharmacophore features are color-coded: green, two aromatic features (F1 and F4); cyan, one hydrogen-bond acceptor feature (F2); purple, one hydrogen-bond donor feature (F3).

The flowchart of virtual screening used in this study is displayed in Figure 2. A total of 35,000 compounds from an in-house database (Niu et al., 2015; Zhou, Tang et al., 2019) were filtered by pharmacophore mapping and molecular docking. All the compounds were imported into MOE to generate a 3D database and used for further virtual screening with the pharmacophore model. For pharmacophore mapping results, the root of the mean square distance (RMSD) between the query features and their matching ligand annotation points is a criterion for evaluating the degree of mapping between the identified compounds and their protein targets (Zhou, Tang et al., 2019). Through the detection of pharmacophore features, 1269 compounds were mapped onto the pharmacophore model and 55 compounds were retrieved from the 1269 molecules with a criterion of RMSD value <0.3 Å (lower values indicate better pharmacophore mapping). To reduce false positive compounds, the 55 compounds were further docked into SARS CoV-2 PLpro-active site by using the docking protocol of MOE. Then, we used a −11 kcal/mol cutoff in docking score to prune the hit list (lower values suggest better binding affinity). The docking results suggested that the top 4 hit compounds showed lower docking scores (below −11.4 kcal/mol) and better pharmacophore mapping (Figure 3). Finally, the 4 candidate compounds (hits 1–4) were selected for the subsequent biological evaluation. The mapping analysis of four hit compounds onto the PLpro-model suggested that aromatic rings of each compound matched the F1 and F4 aromatic features of PLpro-model, respectively (Figure 3). In addition, the oxygen atom of each compound mapped the F2 hydrogen-bond acceptor feature, and its nitrogen atom matched with the F3 hydrogen-bond donor feature of the PLpro-model. These results indicate four hit compounds match well with the pharmacophore features of the PLpro-model.

### In Silico ADMET Studies

One of the important factors in drug discovery is ADME properties (Debnath et al., 2020). ADME estimation is used to predict the pharmacokinetic properties of the selected hits such as absorption, distribution, metabolism, and excretion. According to the physicochemical and pharmacokinetic properties of drug-like compounds, a molecule will be considered a drug-like compound when molecular weight (mol_MW) is <500, the number of hydrogen bond donors (nHD) is <5.0, the number of hydrogen bond acceptors (nHA) is <10, and octanol-water partition coefficient (logP) should be <6.0. The important ADME properties including mol_MW, nHA, nHD, logP, MDCK, HIA, VD, CYP2D6, CYP2C9, T_1/2_, etc. lie within the acceptable ranges and are presented in Table 2. These ADME properties of all the selected hits are in the acceptable range and, therefore, all the selected hits are drug-like molecules.

**TABLE 2:** Predicted ADME descriptors for the selected hits.

Property	Hit compounds				References range
	1	2	3	4	
mol_MW	426.24	463.19	366.14	455.22	Optimal: < 500
nHA	5	6	4	4	Optimal: < 10
nHD	4	3	2	2	Optimal: < 5
logP	4.480	4.951	4.144	5.527	Optimal: < 6
MDCK	4.0e-6	1.7e-5	2.4e-5	8.0e-6	High permeability: > 2e-5
					Medium permeability: 2e-6∼2e-5
					Low permeability: < 2e-6
HIA	0.014	0.006	0.004	0.003	Excellent absorbance: closer to 0
VD (L/kg)	2.847	3.043	0.980	3.286	Proper: 0.04–20
CYP2D6	0.870	0.862	0.620	0.924	Hinder the metabolism: closer to 1
CYP2C9	0.745	0.911	0.869	0.837	Hinder the metabolism: closer to 1
T_1/2_	0.081	0.014	0.056	0.030	Excellent: 0–0.3

mol_MW: mol_molecular weight; nHA: number of hydrogen bond acceptors; nHD: number of hydrogen bond donors; nRot: number of rotatable bonds; logP: the logarithm of the n-octanol/water distribution coefficient; MDCK: apparent permeability coefficient (Madin-Darby Canine Kidney cells); HIA: human intestinal absorption; VD: volume distribution; T_1/2_: half-life.

### Molecular Dynamics Simulations

**FIGURE 4:**
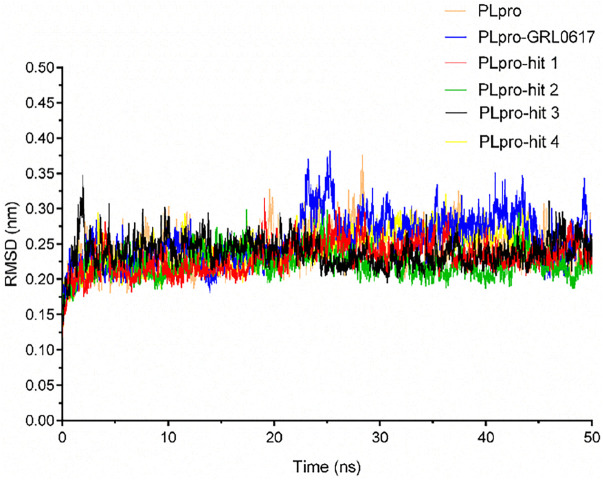
Stability parameter of PLpro alone and PLpro in complex with GRL0617, hit 1, hit 2, hit 3, and hit 4 during 50 ns simulations. Backbone root mean square deviation (RMSD) of MD simulated complexes over the time scale of 50 ns.

**FIGURE 5:**
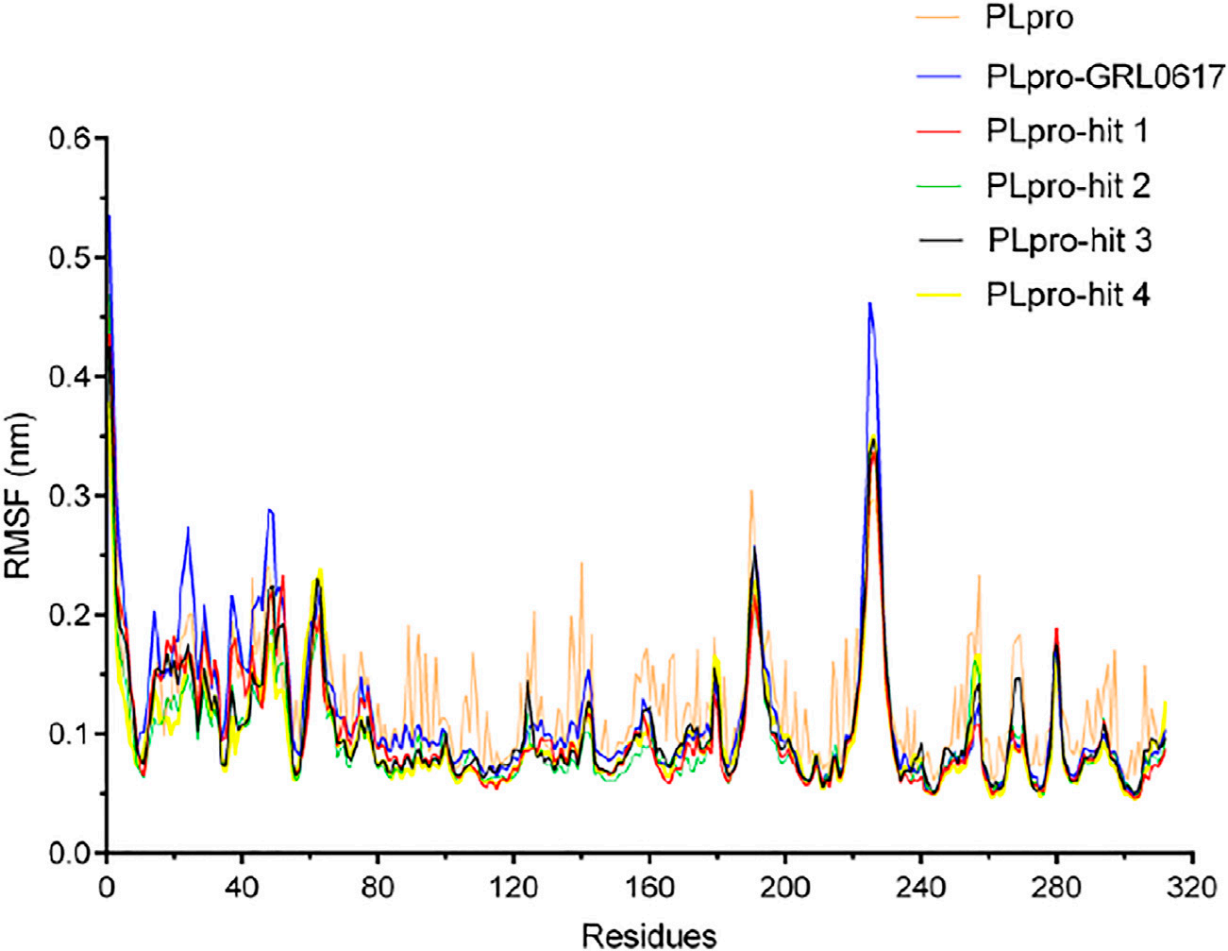
The root mean square fluctuation (RMSF) of PLpro alone and PLpro complex with GRL0617, hit 1, hit 2, hit 3, and hit 4 over the time scale of 50 ns.

To check the stability of PLpro alone and PLpro-ligand complexes, we performed the MD simulations to analyze stability parameters of PLpro alone and PLpro in complex with GRL0617, hit 1, hit 2, hit 3, and hit 4: root mean square deviation (RMSD) and root mean square fluctuation (RMSF) (Dehury, Somavarapu et al., 2020; Dehury, Raina et al., 2020). As shown in Figure 4, the RMSD value of PLpro-GRL0617 complex reached equilibrium at ∼24 ns and remained stable thereafter, while the RMSD value of four PLpro-hit complexes also reached stability at ∼24 ns, and the RMSD value at the final stability was similar to that of PLpro-GRL0617. This shows that the structure of the four PLpro-hit complexes can remain stable throughout the simulation period. The RMSF value is an important parameter that yields data about the structural adaptability of every residue in the system. As shown in Figure 5, the RMSF values for all the residues in the PLpro alone and PLpro-ligand complexes were calculated. We found that compared with PLpro alone, the RMSF fluctuation values of the conserved amino acids (Gln269, Asp164, Tyr264, Leu162, Pro248, and Pro247) in PLpro-hits and PLpro-GRL0617 were very low, suggesting that these molecules could form stronger interactions with these conserved residues. Based on the above analysis, we can conclude that these four selected hits can bind stably to the binding pocket of PLpro.

### Dynamics of Secondary Structure Elements and Inter-Molecular Hydrogen Bonds During Molecular Dynamics

**FIGURE 6:**
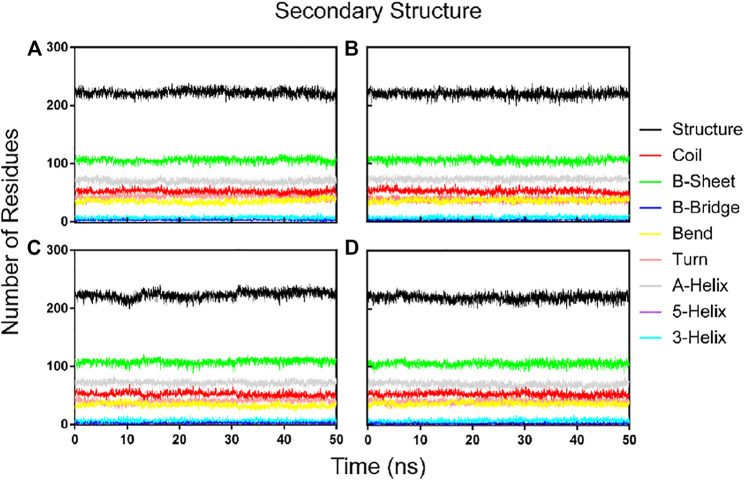
Dynamics of evolution of secondary structure elements of the PLpro complex with hit 1 **(A)**, hit 2 **(B)**, hit 3 **(C)**, and hit4 **(D)** during the MD simulations.

**FIGURE 7:**
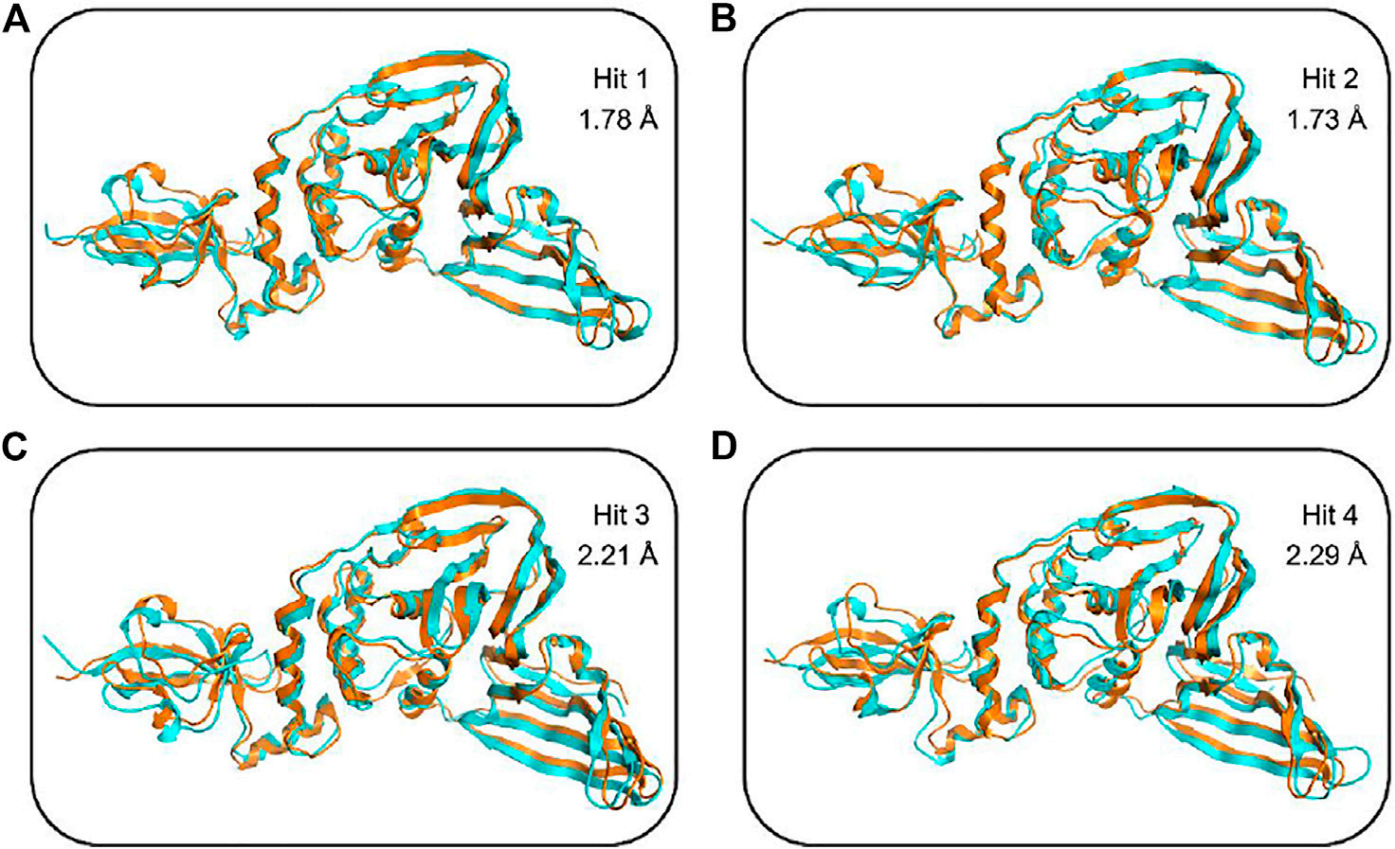
Structural superimposition of the top ranked cluster representative of PLpro-hit complexes (shown in cyan) obtained from MD simulation as compared to the PLpro alone (orange) without any ligand. **(A)** Secondary structure changes of PLpro protein upon binding to hit 1. **(B)** Secondary structure changes of PLpro protein upon binding to hit 2. **(C)** Secondary structure changes of PLpro protein upon binding to hit 3. **(D)** Secondary structure changes of PLpro protein upon binding to hit 4.

**FIGURE 8:**
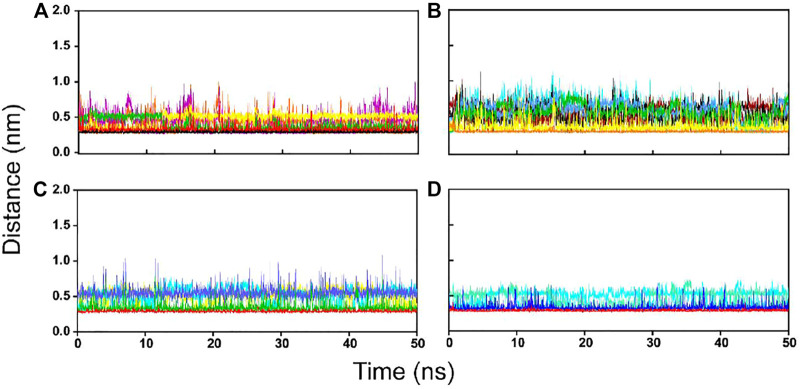
The inter-molecular hydrogen bond dynamics of PLpro-hit complexes during the MD simulation. **(A)** Hit 1. **(B)** Hit 2. **(C)** Hit 3. **(D)** Hit 4.

To further confirm the stability of the PLpro-hit complex systems, we also monitored the changes of secondary structures upon binding to hits during MD simulations using the DSSP algorithm (Figure 6). The results indicated that there were no significant changes in structural elements such as α-helical and β-sheet content observed during the entire simulation time, which further confirmed the stability of our studied systems. To obtain the structural representative from simulation systems, we performed ensemble RMSD based cluster analysis. Based on the RMSD from structural ensembles, the representative snapshot of each PLpro-hit complex was superimposed with the PLpro alone (orange). As shown from Figure 7, it can be clearly observed that the PLpro complexes superpose well with the PLpro alone with a Cα-RMSD < 2.5 Å, indicating that all systems retained the structural integrity and maintained the same fold with minor changes in the PLpro of SARS-Cov-2. In addition, we also measured the inter-atomic distance profile of the important interacting atom pairs (hydrogen bond: for details see Table 3) of the PLpro and hits during the MD simulation (Figure 8).

**TABLE 3:** Hydrogen bond and its occupancy formed in the binding of each hit and PLpro during the MD simulation.

Acceptor	Hydrogen	Donor	Occupancy %
**Hit 1**			
Hit 1@O	Gln269@H	Gln269@N	99.6
Hit 1@N1	Gln269@HE21	Gln269@NE2	75.0
Asp164@OD1	Hit 1@H	Hit 1@N	15.8
Asp164@OD2	Hit 1@H	Hit 1@N	41.7
Glu167@OE1	Hit 1@H21	Hit 1@N2	32.7
Glu167@OE2	Hit 1@H21	Hit 1@N2	27.9
**Hit 2**			
Hit 2@O	Gln269@H	Gln269@N	98.4
Hit 2@N1	Gln269@HE21	Gln269@NE2	50.8
Asp164@OD1	Hit 2@H	Hit 2@N	4.4
Asp164@OD2	Hit 2@H	Hit 2@N	6.6
Asp164@OD1	Hit 2@H20	Hit 2@N2	1.8
Asp164@OD2	Hit 2@H20	Hit 2@N2	3.0
Glu167@OE1	Hit 2@H20	Hit 2@N2	6.8
Glu167@OE2	Hit 2@H20	Hit 2@N2	5.9
**Hit 3**			
Hit 3@O	Gln269@H	Gln269@N	99.3
Hit 3@N1	Gln269@HE21	Gln269@NE2	65.5
Hit 3@O1	Gln269@HE21	Gln269@NE2	0.1
ASP164@OD1	Hit 3@H	Hit 3@N	10.6
ASP164@OD2	Hit 3@H	Hit 3@N	9.9
**Hit 4**			
Hit 4@O	Gln269@H	Gln269@N	99.5
Hit 4@N1	Gln269@HE21	Gln269@NE2	71.8
Asp164@OD1	Hit 4@H	Hit 4@N	33.7
Asp164@OD2	Hit 4@H	Hit 4@N	17.9

### Biological Validation

To validate the inhibitory activities of hits 1-4 against SARS CoV-2 PLpro, the enzyme inhibition assay was performed. As shown in Table 4, all compounds showed excellent inhibition activities toward SARS CoV-2 PLpro with IC_50_ values in the micromolar range (IC_50_ < 3 µM). Among them, the most promising compound, hit 2 is the best PLpro inhibitor and its inhibitory activity (IC_50_ = 0.6 ± 0.2 µM) was about 4 times higher than that of the positive control GRL0617 (IC_50_ = 2.5 ± 0.4 µM). In addition, hit 4 showed about a threefold increase in inhibitory activity (IC_50_ = 0.8 ± 0.3 µM) toward PLpro compared with GRL0617. The results indicated that the two hits are novel and highly potent inhibitors targeting SARS CoV-2 PLpro, making it a good candidate for further optimization toward designing more potent PLpro inhibitors.

**TABLE 4:** Hit compounds selected from an in-house database.

Compounds	Chemical structure	RMSD [Å]	Docking score [kcal/mol]^b)^	IC_50_ [µM]
1	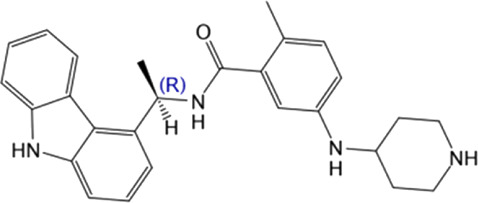	0.2986	−11.41	2.4 ± 0.6
2	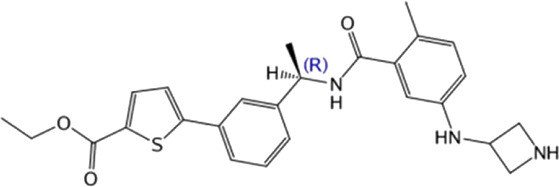	0.2569	−11.54	0.6 ± 0.2
3	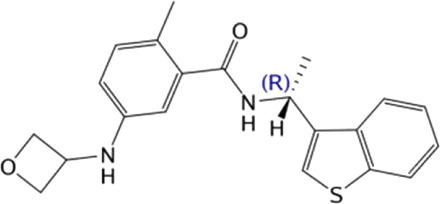	0.2747	−11.43	2.1 ± 0.5
4	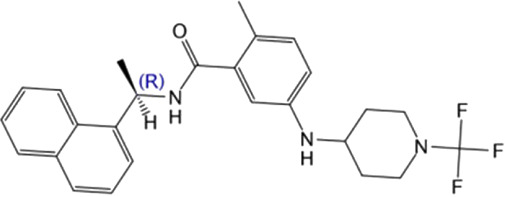	0.2557	−11.52	0.8 ± 0.3
GRL0617				2.5 ± 0.4

### Binding Mode Analysis

**FIGURE 9:**
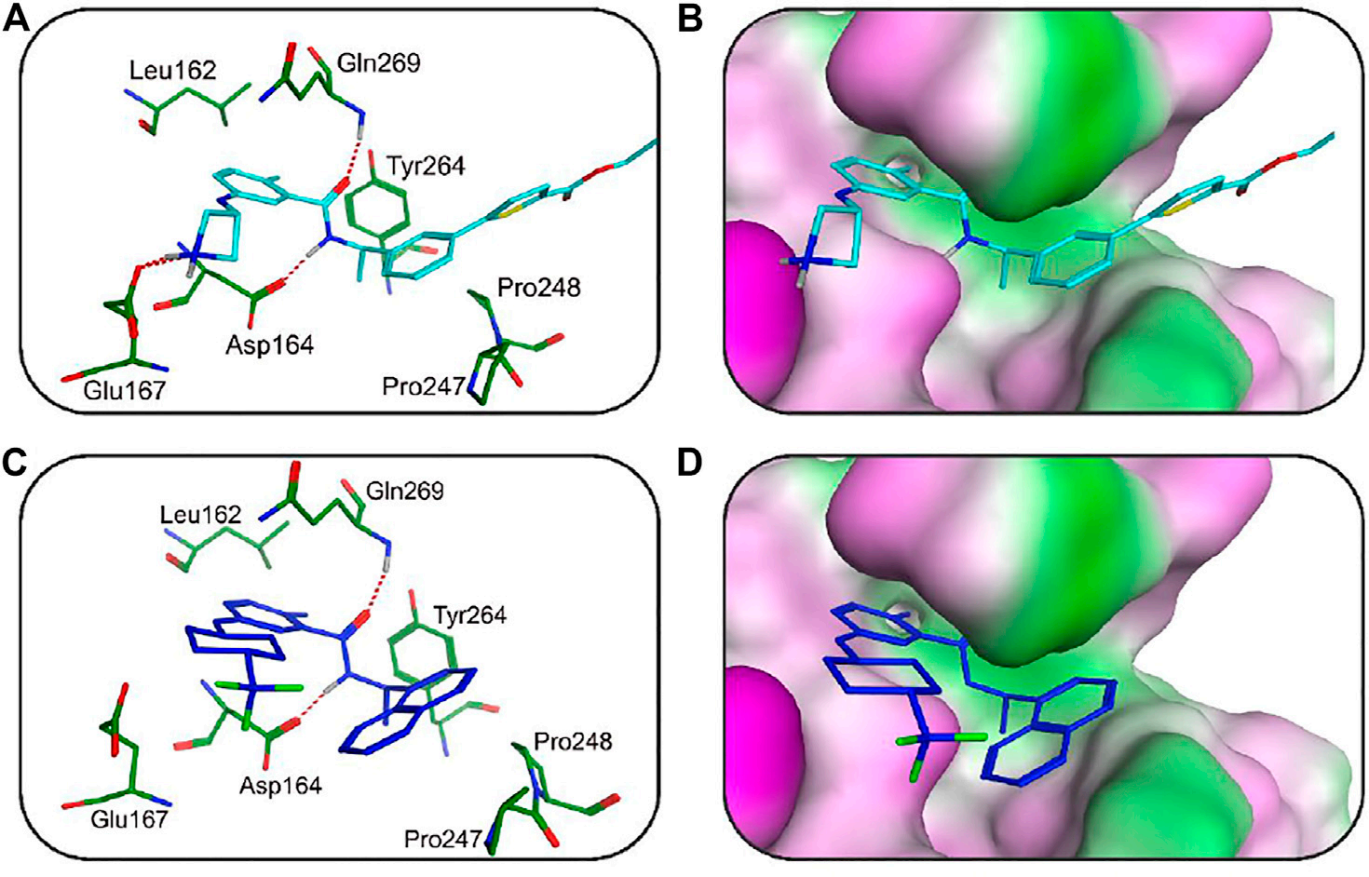
**(A)** The 3D ligand–protein interaction diagram for the binding site of the SARS CoV-2 PLpro with hit 2. The active-site residues are shown in green stick form. Hit 2 is color-coded by cyan. The hydrogen-bond network with protein residues is represented by dotted lines. **(B)** Closeup stereo view of the structure of the PLpro-hit 2 complex. **(C)** The 3D ligand–protein interaction diagram for the binding site of the SARS CoV-2 PLpro with hit 4. Hit 4 is color-coded blue. The active-site residues are shown in green stick form. The hydrogen-bond interaction is represented in dotted lines. **(D)** Closeup stereo view of the structure of the PLpro-hit 4 complex.

To further predict the molecular binding mechanism of hit 2 and 4 with PLpro, the two hits were docked into the PLpro-active site via the docking protocol from MOE. As shown in Figures 9A,B, hit 2 occupied a wide groove of PLpro surrounded by some key residues such as Gln269, Asp164, Tyr264, Leu162, Pro248, and Pro247. The amide group of hit 2 formed two hydrogen-bond interactions with Gln269 and Asp164 while the nitrogen atom of the azetidine group established a hydrogen-bond interaction with Glu167. In addition, the hydrophobic aromatic groups of hit 2 were engaged in hydrophobic interactions with Tyr264, Leu162, Pro248, and Pro247. As illustrated in Figures 9C,D, it is observed that hit 4 formed hydrogen-bond interactions with Gln269 and Asp164. Like hit 2, the hydrophobic aromatic groups of hit 4 also formed hydrophobic interactions with Leu162, Tyr264, Pro247, and Pro248. The docking results suggested that the two hits could have the necessary interactions with key active-site residues of PLpro. From closeup stereo view of the structure of the PLpro-ligand complex, the chemical structure of hit 2 can fit better with the active pocket of PLpro than that of hit 4, indicating the better inhibitory activity of hit 2.

## Conclusion

PLpro is one of the most promising druggable targets of SARS CoV-2. This study reported the identification of four novel and potent inhibitors of SARS CoV-2 PLpro through a combined screening approach of pharmacophore mapping and molecular docking studies. These hits showed excellent inhibitory activities against PLpro with IC_50_ values ranging from 0.6 to 2.4 μM. Particularly, hit 2 and 4 significantly inhibited PLpro in the low micromolar range with IC_50_ < 1 µM. Further chemical optimization of hit 2 and 4 can lead to the highly selective and potent PLpro inhibitors with potential for the treatment of COVID-19. Furthermore, the approach applied here can also offer a guideline for screening of other molecular databases to discover more novel PLpro inhibitors with unknown structures in the future.

## Data Availability

The original contributions presented in the study are included in the article/Supplementary Material, further inquiries can be directed to the corresponding authors.
